# Two laser-assisted hatching methods of embryos in ART: a systematic review and meta-analysis

**DOI:** 10.1186/s12884-024-06380-8

**Published:** 2024-04-22

**Authors:** Kexin Chen, Mengying Gao, Yao Wu, Zhixin Hu, Lu Tang, Minyao Li, Mei Tian, Hao Cui, Yanrong Huang, Youzhen Han, Lei Li, Yonggang Li, Yunxiu Li, Ze Wu, Zouying Tang, Ronghui Zhang, Yuerong Wu, Yizhi Zhang, Yan Guo, Hongqing Zhang, Lifeng Xiang, Jiacong Yan

**Affiliations:** 1 Department of Reproductive Medicine, NHC Key Laboratory of Healthy Birth and Birth Defect Prevention in Western China, First People’s Hospital of Yunnan Province, Kunming, China; 2 KUST-YPFPH Reproductive Medicine Joint Research Center, Kunming, Yunnan China; 3 Kunming Maternity and Child Care Hospital, Kunming, Yunnan China; 4 The First People’s Hospital of Qujing, Qujing, Yunnan China

**Keywords:** Drilling laser-assisted hatching, Thinning laser-assisted hatching, Clinical pregnancy, ART, Zona pellucida

## Abstract

**Background:**

Laser-assisted hatching (LAH) stands as the predominant technique for removing the zona pellucida (ZP) in embryos, primarily consisting of two methods: drilling laser-assisted hatching (D-LAH) and thinning laser-assisted hatching (T-LAH). Presently, both methods have limitations, and their comparative efficacy for embryo implantation and clinical pregnancy remains uncertain.

**Aim:**

Evaluate the impact of D-LAH and T-LAH on clinical pregnancy rates within assisted reproductive technology (ART).

**Methods:**

We systematically searched electronic databases including PubMed, Web of Science, and Cochrane Library until July 20, 2022. This study encompassed observational studies and randomized controlled trials (RCTs). A 95% confidence interval (CI) was utilized for assessing the risk ratio (RR) of pregnancy outcomes. The level of heterogeneity was measured using I^2^ statistics, considering a value exceeding 50% as indicative of substantial heterogeneity.

**Results:**

The meta-analysis scrutinized 9 studies involving 2405 clinical pregnancies from D-LAH and 2239 from T-LAH. Findings suggested no considerable variation in the clinical pregnancy rates between the two techniques (RR = 0.93, 95% CI: 0.79–1.10, I^2^ = 71%, *P* = 0.41). Subgroup analyses also revealed no substantial differences. However, D-LAH exhibited a notably higher occurrence of singleton pregnancies compared to T-LAH (RR = 2.28, 95% CI: 1.08–4.82, I^2^ = 89%, *P* = 0.03). There were no noteworthy distinctions observed in other secondary outcomes encompassing implantation rate, multiple pregnancies, ongoing pregnancy, miscarriage, premature birth, and live birth.

**Conclusion:**

Both the primary findings and subgroup analyses showed no marked variance in clinical pregnancy rates between D-LAH and T-LAH. Therefore, patients with varying conditions should select their preferred LAH technique after assessing their individual situation. However, due to the restricted number of studies involved, accurately gauging the influence of these laser techniques on clinical outcomes is challenging, necessitating further RCTs and high-quality studies to enhance the success rate of ART.

**Trial registration:**

PROSPERO: CRD42022347066.

**Supplementary Information:**

The online version contains supplementary material available at 10.1186/s12884-024-06380-8.

## Introduction

Successful blastocyst hatching is critical for embryo implantation during development. Early embryos are enclosed by the zona pellucida (ZP), a cell-free membrane that measures 13 ~ 15 mm [1]. As the in vitro culture time of embryos extends, the density of the ZP increases [2]. If the embryo fails to detach from the ZP or if the ZP undergoes abnormal development, it may result in the failure of embryo implantation [3]. To facilitate successful embryo hatching, assisted hatching (AH) is a technique employed in assisted reproductive technology (ART) [4, 5]. AH entails the manual creation of an aperture in the ZP of the embryo to facilitate the hatching process [6]. The effect of AH on the live birth rate remains uncertain at present [3, 7]. Lacey et al.'s systematic review and meta-analysis aimed to evaluate AH's impact on ART outcomes; however, the study's results did not offer conclusive evidence regarding its effect on live birth rates [8]. Although AH might enhance clinical pregnancy rates, the current research articles lack quality, demanding further high-quality studies for definitive conclusions [7]. Presently, AH's impact on ART remains unclear, potentially influenced by varying AH methods adopted by individual reproductive centers or differences in operational procedures.

These techniques encompass acidified Tyrode's solution/medium, mechanical intervention, and laser-assisted hatching (LAH) on the ZP [9, 10]. However, the chemical-based method carries the risk of potential ZP damage and adverse effects on embryonic development, especially when handling large sample batches [11]. On the other hand, the mechanical approach necessitates considerable expertise and consumes time, presenting challenges in implementation [11]. LAH is the most widely used AH method, and its various techniques also affect ART outcomes [12]. It serves as a preferred choice for separating embryos from the ZP due to its simplicity, rapid operation, precise laser application, and minimal disruption to embryos, among other benefits [13]. Significantly, LAH appears more effective in enhancing pregnancy rates than chemical acidification [14]. Furthermore, frozen embryos subjected to LAH exhibit notably higher live birth rates than those no-LAH [15].

Currently, two primary methods are utilized in clinical LAH procedures: thinning and drilling. Thinning laser-assisted hatching (T-LAH) involves laser removal of the outer layer of the ZP, leaving the inner layer intact. Drilling laser-assisted hatching (D-LAH) aims to completely penetrate both ZP layers, resulting in a single membrane opening [16, 17]. Nevertheless, both techniques have limitations. D-LAH might cause blastomere loss in embryos under high nutrient and antibiotic exposure, hampering blastocyst development [18]. Furthermore, D-LAH has the potential to lead to the creation of monozygotic twins by means of blastomeres drilling [19]. The study found that D-LAH had a higher hatching rate than T-LAH in mouse blastocysts, but there was no significant difference in blastocyst formation rate [20]. Conversely, T-LAH, considered less harmful to embryos, could impede the in vitro hatching process based on research involving mouse embryos [21]. Despite the prevalence of both D-LAH and T-LAH in medical practice, determining the superior method remains contentious [22]. Existing studies present conflicting findings: some assert D-LAH's superiority, some favor T-LAH, while others report no substantial disparity [12, 23, 24].

The objective of this study is to scrutinize the impact of T-LAH and D-LAH on clinical pregnancies and associated outcomes through a systematic review and meta-analysis, aiming to offer valuable theoretical insights for clinical methodologies. This study enrolled patients undergoing in vitro fertilization (IVF) or intracytoplasmic sperm injection (ICSI) procedures, encompassing various age groups without specific age limitations. A portion of the sample underwent D-LAH, while another underwent T-LAH, allowing a comparison of outcomes such as implantation rate, clinical pregnancy rate, and abortion rate.

### Materials and methods

Following the guidelines outlined in the Preferred Reporting Items for Systematic Reviews and Meta-Analyses (PRISMA), this study performed a thorough analysis employing both quantitative and qualitative methodologies [25].

### Literature search

**Fig. 1:**
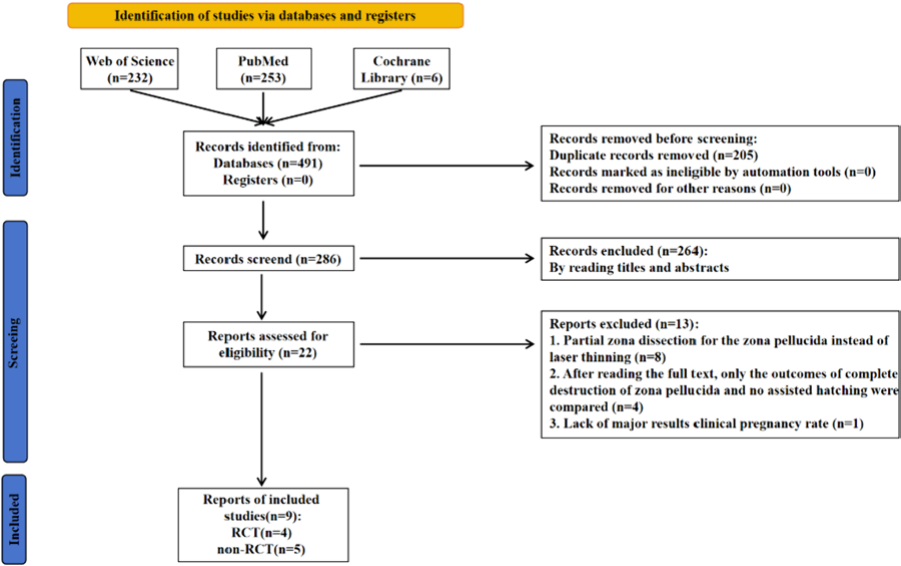
PRISMA flow chart

Electronic sources, including PubMed, Web of Science, and Cochrane Library, were reviewed until July 20, 2022. The following medical topic header (MeSH) phrases and/or keywords are primarily used for retrieval: ((assisted hatching) AND (zona pellucida)), ((thinning and drilling) AND (assisted hatching)), ((thinning and opening) AND (assisted hatching)), ((thinning and breaching) AND (assisted hatching)). Two reviewers (C.K. and H.Z.) conducted a literature search that yielded a total of 491 studies. After applying exclusion criteria using EndNote, 209 studies met the requirements for quantitative analysis. These studies were selected based on the reviewers' evaluation of the titles, abstracts, and full texts of the remaining 205 studies [12, 16, 23, 24, 26–30]. The literature search specifically focused on English papers, as depicted in Fig. 1, which outlines the retrieval and inclusion process.

### Inclusion and exclusion criteria

During the literature screening process, the inclusion and exclusion standards for the studies are determined by reading and evaluating their significance. Two reviewers (C.K. and H.Z.) separately filter the literature during the literature screening process, and a third reviewer judges the contentious pieces (Y.J).

Inclusion criteria:

1. The study designs encompass randomized controlled trials (RCTs), non-randomized controlled trials (non-RCTs), and prospective studies.
2. Patients involved in the study experienced at least one failed implantation cycle.
3. LAH involved both drilling and thinning of the ZP.
4. Post-LAH clinical outcomes include, at minimum, achieving clinical pregnancy.

Exclusion criteria:

1. The types of publications considered encompass posters, meetings, letters, comments, and editorials. Publications in languages other than English were omitted.
2. AH techniques involved either chemical acidification or mechanical methods.
3. The outcomes assessed post AH specifically focused on blastocyst formation rate and implantation rate.

### Data extraction

To avoid overlooking relevant research, two evaluators (C.K. and H.Z.) conducted independent studies using specified keywords and MeSH. The evaluators (C.K. and H.Z.) extracted data from the studies, and any contentious findings were reexamined by a third reviewer (Y.J.) before the authors reached a consensus.

### Quality assessment

Two evaluators (C.K. and H.Z.) assessed the quality of the literature, while a third reviewer (Y.J.) resolved any ambiguities. The Cochrane risk-of-bias tool and Newcastle Ottawa Scale (NOS) were selected for quality evaluation since the research included both RCTs and non-RCTs [31]. The Cochrane risk-of-bias tool primarily assesses RCTs, covering elements such as random sequence generation, allocation concealment, blinding of participants and personnel, blinding of outcome assessment, incomplete outcome data, selective reporting, and other biases. On the other hand, the Newcastle–Ottawa Scale (NOS) is tailored for evaluating bias in non-RCTs its main entries include: is the case definition adequate; representativeness of the cases; selection of controls; definition of controls; Comparability of cases and controls on the basis of the design or analysis; ascertainment of exposure; Same method of ascertainment for cases and controls; non-response rate. Publication bias is appraised through funnel charts.

### Statistical analysis

Review Manager version 5.4 (The Cochrane Collaboration) was used for meta-analysis. Both main and secondary outcomes are considered in statistical analysis. The risk ratio (RR) of pregnant result was examined using a 95% confidence interval (CI). Heterogeneity was measured by I^2^. The study is regarded as extremely heterogeneous when I^2^ > 50% [32]. Given the diverse population sources in each study resulting in considerable variability, the random-effects model was chosen for analysis.

## Results

### Search results and basic characteristics

A comprehensive search was conducted across PubMed, Web of Science, and the Cochrane library, resulting in a total of 491 studies. Using EndNote 20, 205 duplicate studies were removed, leaving 286 studies for title and abstract screening. Following this screening process, 264 studies were excluded. The complete texts of the remaining 22 studies were thoroughly read, resulting in the selection of 9 studies for qualitative and quantitative analysis. The diagram in Fig. 1 depicted the complete filtering procedure. Nine studies totaling 4 RCT [16, 28–30] and 5 non-RCT [12, 23, 24, 26, 27] were included. The particular information traits that were examined are listed in Table 1.

**Table 1:** Specific information characteristics

Study/ Country	Method for allocation	Laser assisted hatching system	Inclusion criteria	Exclusion criteria	Age	Times of AH	Embryo (fresh or frozen)	Participants D-LAH/T-LAH
E.Mantoudis et al. 2001 (United Kingdom) [26]	non-RCT	Fertilase™, Medical Technologies, Montreux SA, Switzerland	(i) Patients having frozen embryo replacement (ii) Two previous IVF or ICSI failed cycles (iii) Patients requiring high dose gonadotrophins, more than 50 ampoules, or a dose of 5 or more ampoules per day	Not stated	> 38	Day 3	Fresh	77/245
TS Ghobara et al. 2006 (United Kingdom) [27]	non-RCT	MMT Medical Technologies, Montreux SA, Switzerland	(i) The woman’s age was 38 years or more (ii) The couple had had three or more unsuccessful IVF/ICSI cycles	Not stated	27–48	Not stated	Fresh	312/592
Ernest Hung Yu Ng et al. 2008 (China) [28]	RCT	Zona Knife; SL Microtest GmbH, Jena,Germany	Patients had at least two frozen embryos available for transfer	(i) More than three stimulated IVF cycles (ii) Only one frozen embryo before thawing (iii) Frozen embryos replaced in stimulated IVF cycles (iv) Recipients of donor oocytes (v) Lysis of all frozen embryos on thawing (vi) Frozen embryos with ZP thickness of < 13 mm	Not stated	Cleavage stage	Frozen	90/90
B. Ma et al. 2014 (China) [16]	RCT	Lykos laser: Hamilton Thorne, Beverly, MA, USA	Patients had at least three previous implantation failures in fresh day-3 embryo transfers	Not stated	< 37	Day 3	Fresh	52/49
Minh Tam Le et al. 2018 (Vietnamese) [29]	RCT	Saturn 5 Active; BioMedical Instruments, Zoellnitz, Germany	(i)A maximum of three previous failed IVF-ET procedures (ii)Undergoing FET	Not stated	22–47	Day 3	Frozen	85/86
Jung-Woo Lee et al. 2019 (Korea) [24]	non-RCT	Hamilton Thorne Instruments Biosciences, Beverly,MA, USA	(i)Patients had a least two episodes of implantation failure (ii)Patients had an endometrial thickness ≥ 8 mm on the day of embryos transfer	Patients who underwent oocyte donation, oocyte activation, or genetic diagnosis, as well as those who used surrogate mother	< 38, ≥ 38	Cleavage stage	Fresh	218/191
Chengjun Liu et al. 2020 (China) [23]	non-RCT	HAMILTON THORNE, ZILOS-tk, USA	(i) Frozen blastocyst transfer with LAH (ii) The first frozen embryo transfer (FET) cycle and no fresh embryo transfer cycle in our center (iii) Normal karyotype of both husband and wife (iv) Endometrial thickness greater than 7 mm on the day of FET	(i) Female with endometriosis or adenomyosis (ii) Unexplained infertility of the couples	31.1 ± 4.2	Day 3	Frozen	864/254
Yujiang Wang et al. 2022 (China) [12]	non-RCT	ZILOS-tk®, Hamilton-Thorne Instruments Biosciences	(i) Patients had undergone Day 4 frozen-thawed embryo transfer (ii) Patients had a maximum of four previous failed IVF-ET procedures (iii) All embryo transfers had been performed using embryos at the morula or cleavage-stage (blastomeres continued to grow after thawing)	Not stated	22–47	Day 4	Frozen	694/716
Ling Zhang et al. 2022 (China) [30]	RCT	MTG Medical Technology, Altdorf, Germany	(i) Couples with more than 2 highly fragmented day-3 cleavage embryos (specified as embryos originating from 2PN zygote, with fragment rate > 25% and at least 4 blastomeres) (ii) Receiving extended in vitro culture	(i) Abnormal karyotypes of any partner (ii) Embryos originating from assisted oocyte activating or in vitro maturation procedure (iii) Familial infertility of any partner	< 40	Day 3	Fresh	13/16

### Quality assessment of the included studies

In this meta-analysis, we conducted an evaluation of 4 RCTs using the Cochrane risk-of-bias assessment. We assessed various sources of bias including selection bias, performance bias, detection bias, attrition bias, reporting bias, and other potential biases by examining key factors such as random sequence generation, allocation concealment, blinding of participants and personnel, blinding of outcome assessment, incomplete outcome data, and selective reporting. The results indicated a relatively low and unclear risk (Supplementary Fig. S1a). We used NOS to evaluate the risk of 5 non-RCTs, and all included studies scored ≥ 4, indicating medium-quality studies (Supplementary Fig. S 1b).

## Main outcome

### Clinical pregnancy

**Fig. 2:**
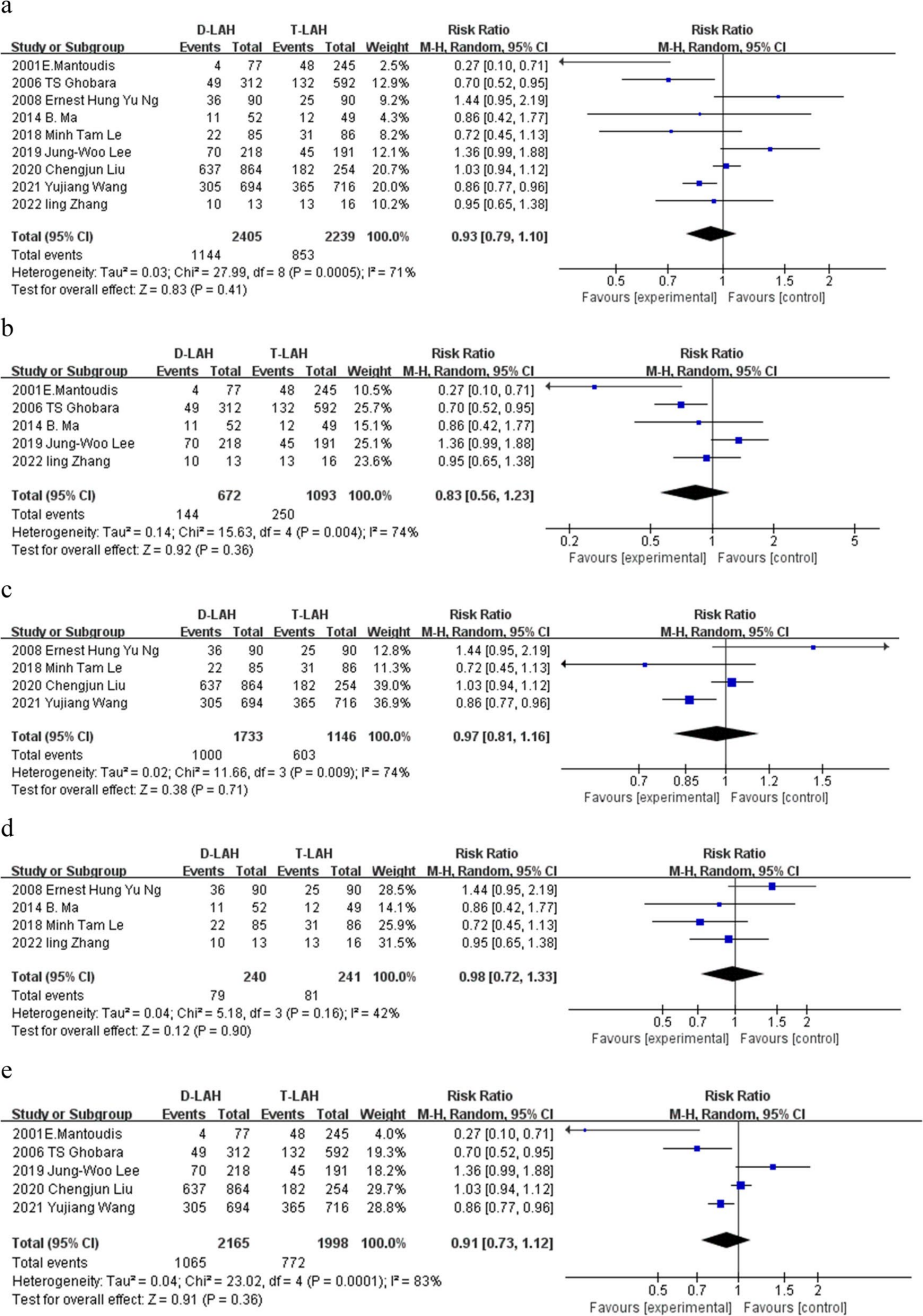
Clinical pregnancy.The forest plot of D-LAH and T-LAH clinical pregnancy. **a** A total of 9 studies were included in this meta-analysis showing that there was no significant difference between D-LAH and T-LAH in clinical pregnancy. **b**-**e** The subgroup analysis, fresh embryo, frozen embryo, RCT studies and non-RCT studies in clinical pregnancy that there were no significant difference of two methods. **a** clinical pregnancy; **b** clinical pregnancy (fresh embryo); **c** clinical pregnancy (frozen embryo); **d** clinical pregnancy (RCT studies); **e** clinical pregnancy (non-RCT studies)

Clinical pregnancy included a total of 9 trials. The outcome revealed no discernible variation between D-LAH and T-LAH (RR = 0.93, 95% Cl: 0.79—1.10, I^2^ = 71%, *P* = 0.41, Table 2 and Fig. 2a). We performed a subgroup study on the blastocysts for auxiliary hatching, both fresh and frozen, and the findings revealed no discernible differences between D-LAH and T-LAH (5 studies) (RR = 0.83, 95% Cl: 0.56—1.23, I^2^ = 74%, *P* = 0.36, Table 2 and Fig. 2b) (4 studies) (RR = 0.97, 95% Cl: 0.81—1.16, I^2^ = 74%, *P* = 0.71, Table 2 and Fig. 2c). Then we analyzed the RCT (4 studies) (RR = 0.98, 95% Cl: 0.72—1.33, I^2^ = 42%, *P* = 0.90, Table 2 and Fig. 2d) and non-RCT (5 studies) (RR = 0.91, 95% Cl: 0.73—1.12, I^2^ = 83%, *P* = 0.36, Table 2 and Fig. 2e), and the results still had no significant difference.

**Table 2:** The pooled results of meta-analysis and subgroup analyses for main and secondary outcomes of D-LAH and T-LAH

Group	No. of studies	No. of Events/Total	Effect size (RR 95%Cl)	P	I^2^(%)
Clinical pregnancy	9	D-LAH:1144/2405 T-LAH:853/2239	0.93(0.79–1.10)	0.41	71
Fresh embryo clinical pregnancy	5	D-LAH:144/672 T-LAH:250/1093	0.83(0.56–1.23)	0.36	74
Fresh embryo clinical pregnancy	4	D-LAH:1000/1733 T-LAH:603/1146	0.97(0.81–1.16)	0.71	74
RCT clinical pregnancy	4	D-LAH:79/240 T-LAH:81/241	0.98(0.72–1.33)	0.90	42
Non-RCT clinical pregnancy	5	D-LAH:1065/2165 T-LAH:772/1998	0.91(0.73–1.12)	0.36	83
Implantation rate	6	D-LAH:1286/3740 T-LAH:1038/3872	1.02(0.80–1.28)	0.89	89
Singleton pregnancy	2	D-LAH:669/1082 T-LAH:83/445	2.28(1.08–4.82)	0.03	89
Multiple pregnancy	6	D-LAH:145/1216 T-LAH:149/1376	1.25(0.78–2.00)	0.36	41
Ongoing pregnancy	4	D-LAH:122/406 T-LAH:89/383	1.25(0.89–1.77)	0.20	54
Miscarriages	5	D-LAH:66/473 T-LAH:106/583	0.77(0.58–1.03)	0.07	0
Preterm birth	2	D-LAH:84/779 T-LAH:84/802	0.92(0.46–1.84)	0.82	26
Live birth	3	D-LAH:822/1870 T-LAH:539/1562	0.93(0.79–1.09)	0.37	63

## Secondary outcomes

### Implantation rate

**Fig. 3:**
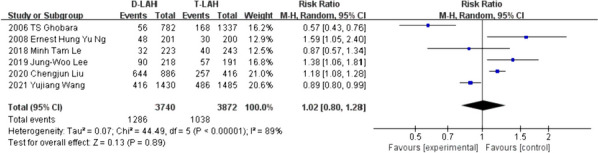
Implantation rate.The meta-analysis included 6 studies comparing D-LAH and T-LAH in terms of implantation rate, and found no significant difference between the two methods. This is displayed in the forest plot

The outcomes of the 6 studies on blastocyst implantation revealed no significant differences between D-LAH and T-LAH. (RR = 1.02, 95% Cl: 0.80—1.28, I^2^ = 89%, *P* = 0.89, Table 2 and Fig. 3).

### Singleton and multiple pregnancies

**Fig. 4:**
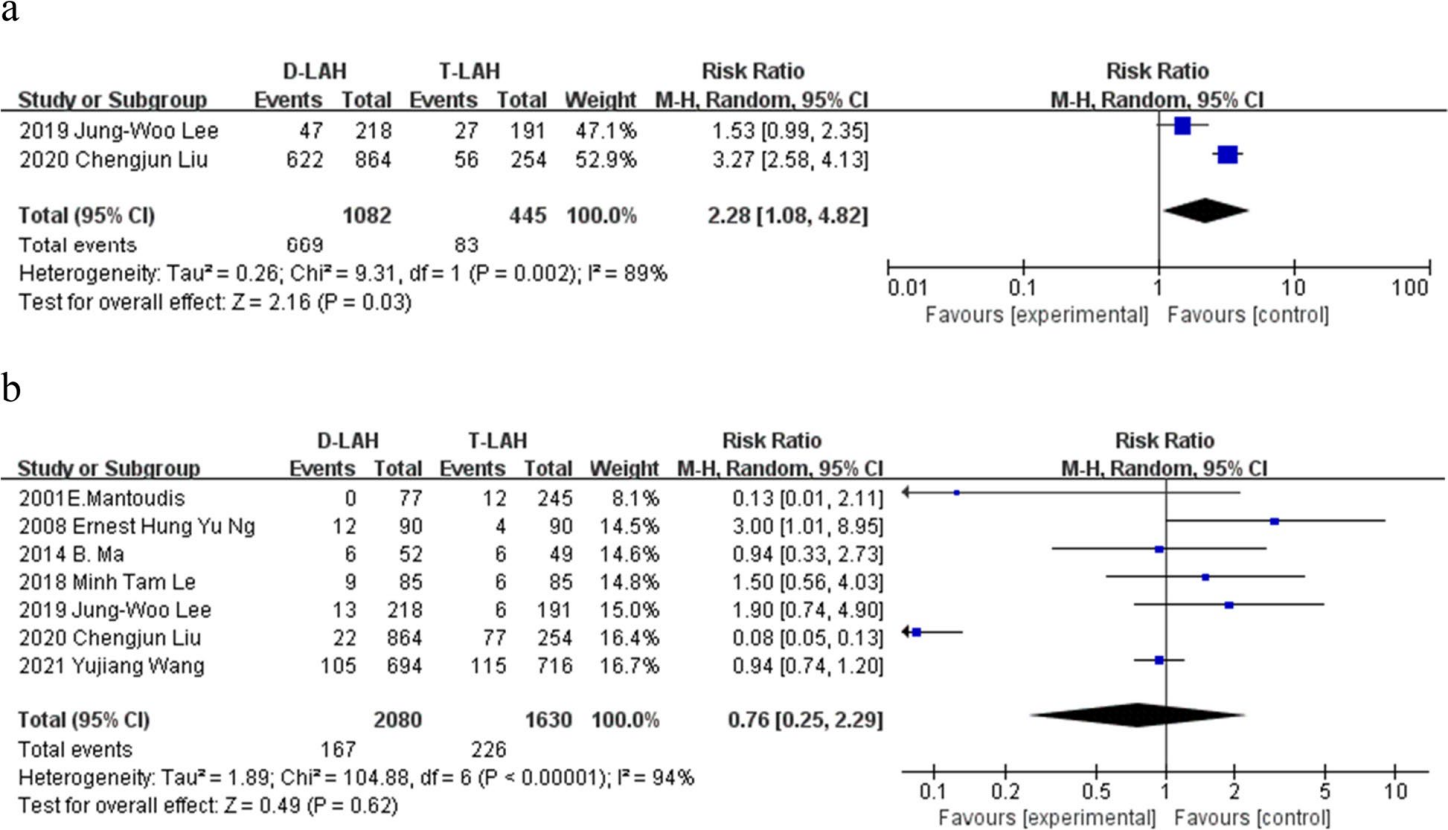
Singleton and multiple pregnancies. The forest plot of D-LAH and T-LAH singleton and multiple pregnancies. **a** 2 studies showing that the singleton pregnancy rate of D-LAH was higher than T-LAH. **b** A total of 7 studies were included in this meta-analysis showing that there was no significant difference between D-LAH and T-LAH in multiple pregnancy. **a** singleton pregnancy; **b** multiple pregnancy

The findings of two singleton pregnancy investigations revealed that D-LAH had a greater singleton pregnancy incidence than T-LAH (RR = 2.28, 95% Cl: 1.08—4.82, I^2^ = 89%, *P* = 0.03, Table 2 and Fig. 4a). However, there was no significant difference in multiple pregnancies (7 studies) (RR = 0.76, 95% Cl: 0.25—2.29, I^2^ = 94%, *P* = 0.62, Table 2 and Fig. 4b).

### Ongoing pregnancy

**Fig. 5:**
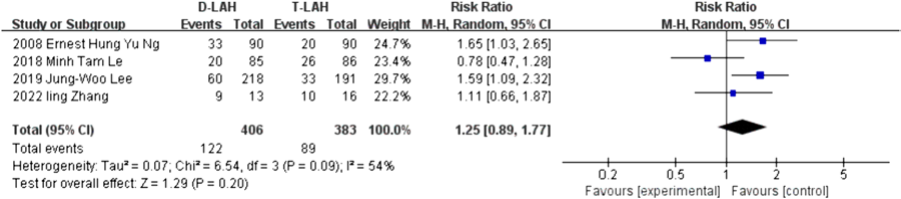
Ongoing pregnancy. The meta-analysis forest plot comparing D-LAH and T-LAH for ongoing pregnancy included 4 studies and found no significant difference between the two procedures

In 4 studies of ongoing pregnancy, the results showed that there was no significant difference between D-LAH and T-LAH (RR = 1.25, 95% Cl: 0.89—1.77, I^2^ = 54%, *P* = 0.20, Table 2 and Fig. 5).

### Miscarriage, premature birth and live birth

**Fig. 6:**
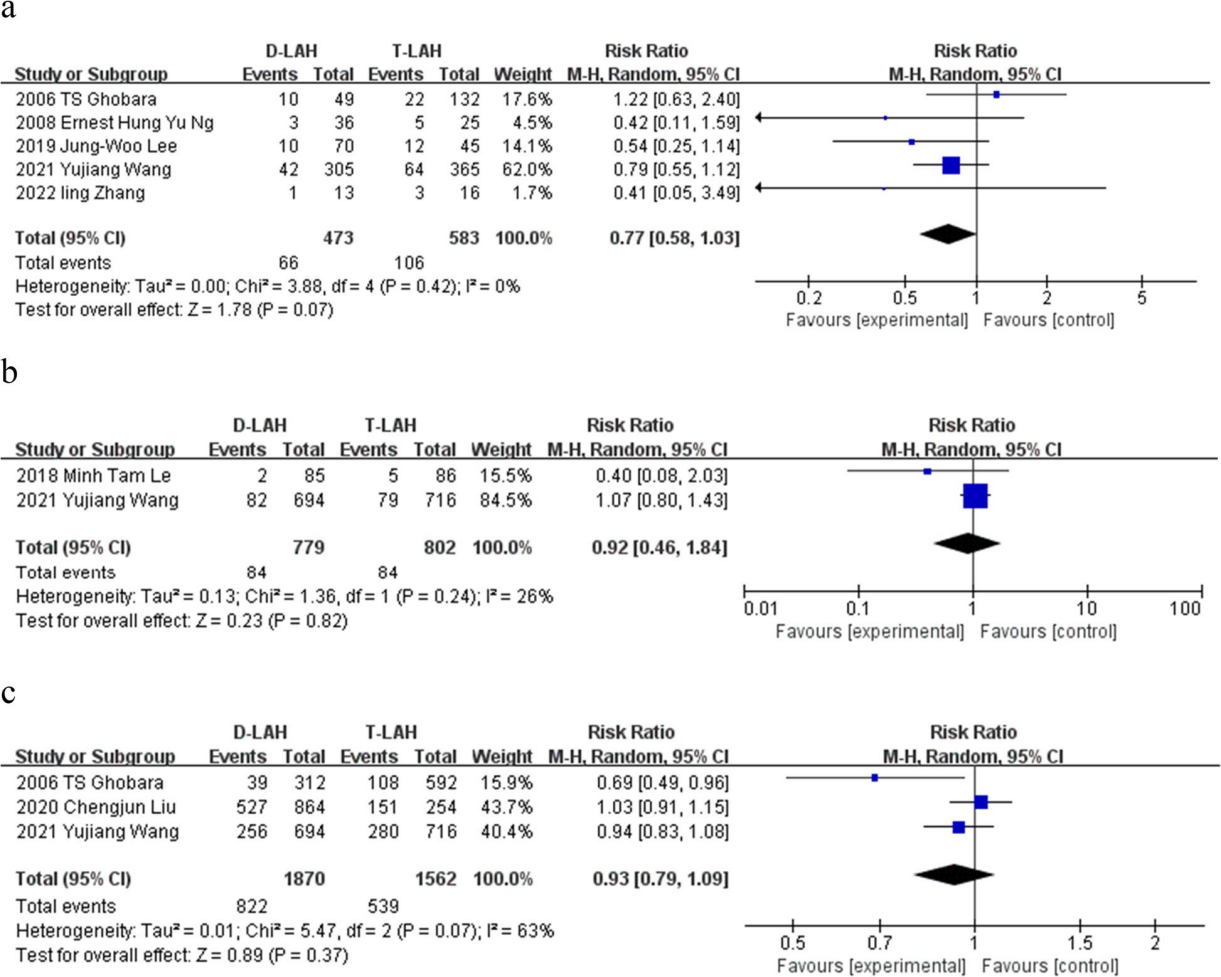
Miscarriage, premature birth and live birth. The forest plot of D-LAH and T-LAH miscarriage, premature birth and live birth. **a** 5 studies were included in this meta-analysis showing that there was no significant difference between D-LAH and T-LAH in miscarriage. However, the miscarriage rate of D-LAH is lower than that of T-LAH. **b**-**c** In the premature birth and live birth, there were no significant difference of two methods. **a** miscarriage; **b** premature birth; **c** live birth

All studies' findings for miscarriage (5 studies), preterm birth (2 studies), and live birth (3 studies) revealed no discernible difference between D-LAH and T-LAH. (RR = 0.77, 95% Cl: 0.58—1.03, I^2^ = 0%, *P* = 0.07, Table 2 and Fig. 6a) (RR = 0.92, 95% Cl: 0.46—1.84, I^2^ = 26%, *P* = 0.82, Table 2 and Fig. 6b) (RR = 0.93, 95% Cl: 0.79—1.09, I^2^ = 63%, *P* = 0.37, Table 2 and Fig. 6c).

## Discussion

### Summary of results

The meta-analysis results showed no significant difference in clinical pregnancy rates between D-LAH and T-LAH for AH. Further subgroup analysis based on fresh or frozen embryos and study type also revealed no significant differences. Overall, the LAH method didn't significantly affect clinical pregnancy outcomes. However, D-LAH showed a higher rate of singleton pregnancies compared to other methods, though no other remarkable distinctions were evident. D-LAH might benefit singleton transplantation, but further research is necessary to validate this. Additionally, we conducted an analysis of multiple pregnancies an initial analysis aimed to assess the heterogeneity of multiple pregnancies. It was found that the study conducted by Chengjun Liu et al. [23] were excluded due to There is a large difference in the number of samples between D-LAH and T-LAH and lack of information regarding patients' abortion history, and the quality of embryo transfer. Consequently, the heterogeneity decreased from 94 to 41%. Nonetheless, there was still no significant distinction observed between D-LAH and T-LAH in terms of their effects (RR = 1.25, 95% Cl: 0.78—2.00, I2 = 41%, *P* = 0.36). There was no significant difference between D-LAH and T-LAH in ongoing pregnancy, miscarriage, preterm birth and live birth. Whether the embryo can be successfully implanted into clinical pregnancy, what is more important is the interaction between mother and fetus, intimal environment, embryo quality and so on [33]. Therefore, LAH is the factor affecting embryonic pregnancy, but it is not the only factor. The heterogeneity analysis of the primary and secondary outcomes is presented in Supplementary Fig. S2.

### Clinical suggestion

No notable differences were found in clinical pregnancy, implantation rate, or live birth between the T-LAH and D-LAH techniques. However, D-LAH notably demonstrated a higher rate of singleton pregnancies than T-LAH. Additionally, following assisted hatching during cleavage, D-LAH showed a greater incidence of blastocyst formation compared to T-LAH [34, 35]. Based on this research, D-LAH may be recommended for clinical use. Nevertheless, considering variations among embryo laboratories and patient populations, the choice of LAH method should align with specific conditions. According to a research by Wang et al. [12], T-LAH had a superior clinical result than D-LAH for patients under 35 with a history of IVF/ICSI failure or 8-10mm endometrial thickness. Additionally, factors such as embryo freezing and freshness, embryo quality, and culture medium were identified to influence ART outcomes [6]. Successful implantation requires synchronized development of both the embryo and endometrium, enabling the expression and secretion of various factors that enhance clinical outcomes by facilitating attachment to the endometrium through the ZP [36]. While D-LAH outperforms T-LAH in singleton pregnancy, no significant differences were observed in other aspects. Therefore, patients with varying conditions should select their preferred LAH technique after assessing their individual situation.

### Advantages and limitations of research

The debate surrounding the two LAH methods for achieving clinical pregnancy persisted [37, 38]. Some studies indicate positive clinical pregnancy outcomes for T-LAH [26], while others report contrary findings or observe no significant differences between the two techniques [23, 24]. This study aimed to evaluate the impact of different LAH techniques on ART outcomes and propose clinical recommendations. Our findings suggest a potential superiority of D-LAH over T-LAH specifically in singleton pregnancies, offering insights for clinical decision-making.

However, the study's low quality necessitates further robust RCT studies for conclusive evidence. However, certain limitations remain in this study. Firstly, the analysis incorporated a limited number of studies. Among the 9 studies evaluating clinical pregnancy outcomes, none explored critical indicators such as blastocyst formation rate, implantation rate, or live birth rate. Subsequent investigations should prioritize assessing the impact on live births. Secondly, significant heterogeneity was identified through a heterogeneity analysis, likely stemming from differences in sample sizes and experimental settings across studies. Thirdly, discrepancies in patient inclusion and exclusion criteria across studies might influence clinical outcomes, considering factors like endometrial thickness, uterine condition, and embryo quality critically impact embryo development and clinical outcomes. Lastly, the study did not address whether assisted reproductive technology contributes to an increased incidence of monozygotic twins, a significant concern in this field. Therefore, more RCTs and high-quality studies are imperative to enhance understanding in this field.

## Conclusion

The meta-analysis revealed no significant difference in clinical pregnancy between D-LAH and T-LAH as the main result. However, secondary results indicated that D-LAH performed better in singleton pregnancy compared to T-LAH. Our findings suggest that D-LAH may offer superior clinical outcomes over T-LAH. Nevertheless, it's crucial to account for potential confounding factors like patient characteristics, blastocyst quality, and study design. To validate these findings, offer clinical recommendations, and improve the success rate of ART, additional high-quality studies and RCTs are imperative.

### Supplementary Information

**Supplementary Material 1.**

## Data Availability

The corresponding author can provide all data for the analyses upon request.

## Abbreviations

ART: Assisted reproductive technology
IVF: In vitro fertilization
ICSI: Intracytoplasmic sperm injection
ET: Embryo transfer
ZP: Zona pellucida
AH: Assisted hatching
LAH: Laser-assisted hatching
T-LAH: Thinning laser-assisted hatching
D-LAH: Drilling laser-assisted hatching
PRISMA: Preferred Reporting Items for Systematic reviews and Meta-Analyses
RCT: Randomized controlled
non-RCT: Non-randomized controlled
NOS: Newcastle Ottawa Scale
RR: Risk ratio
CI: Confidence interval
