# Supplementary material for: Two laser-assisted hatching methods of embryos in ART: a systematic review and meta-analysis
Source: BMC Pregnancy Childbirth. 2024 Apr 22;24:300. doi: 10.1186/s12884-024-06380-8 (PMC11034172; doi:10.1186/s12884-024-06380-8)
Supplement: Supplementary file 1 — Supplementary Material 1. [file 12884_2024_6380_MOESM1_ESM.docx]

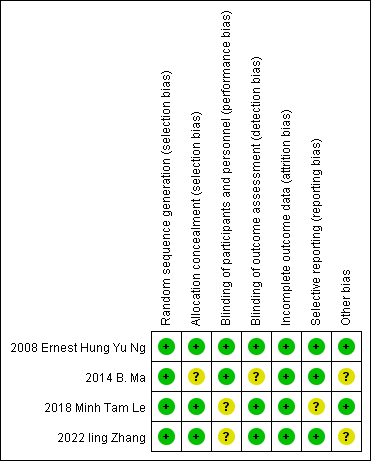
a


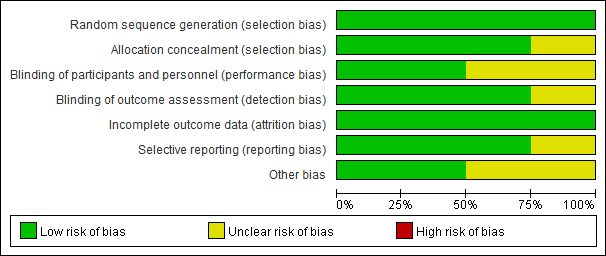


b


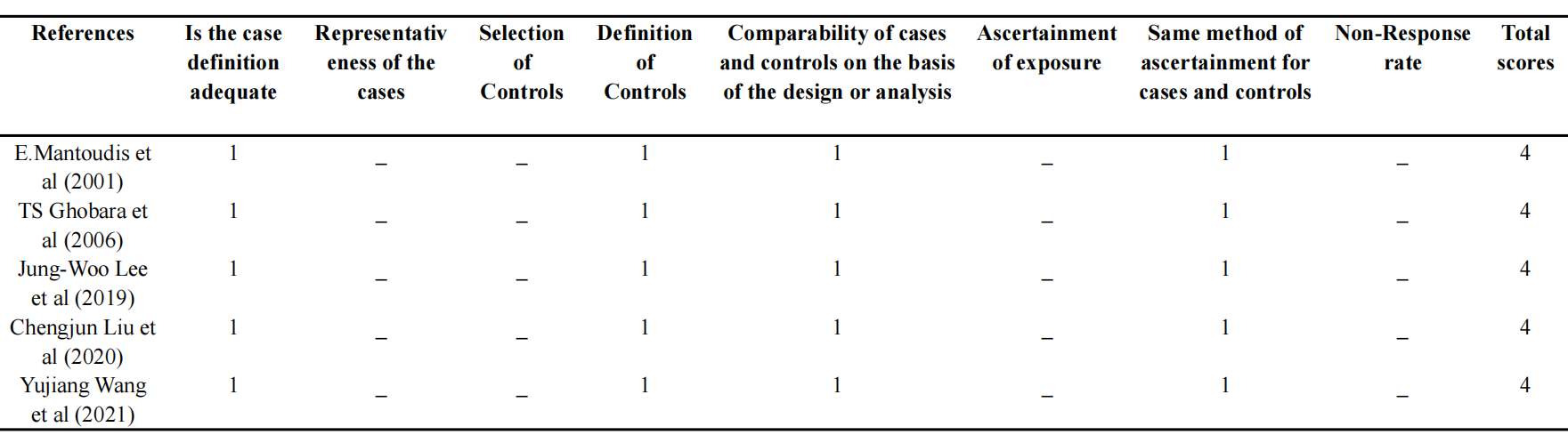


Supplementary Fig. S1: Quality assessment

In this meta-analysis, Cochrane risk-of-bias assessment to evaluate 4 RCT and Newcastle Ottawa Scale (NOS) risk assessment in 5 non-RCT.

(a) Cochrane risk-of-bias assessment; (b) NOS risk assessment.

a


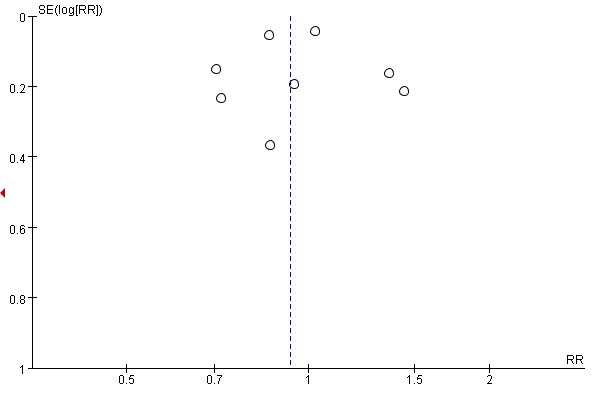


b


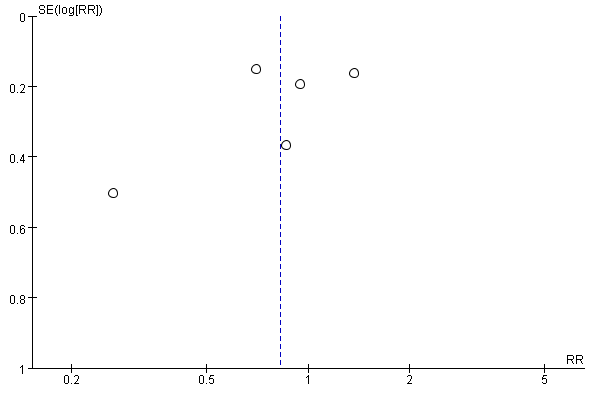


c


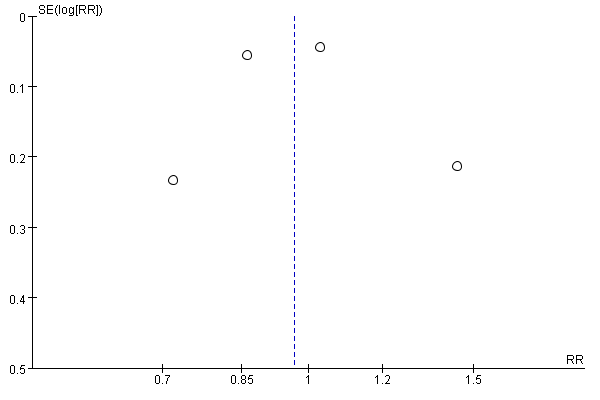


d


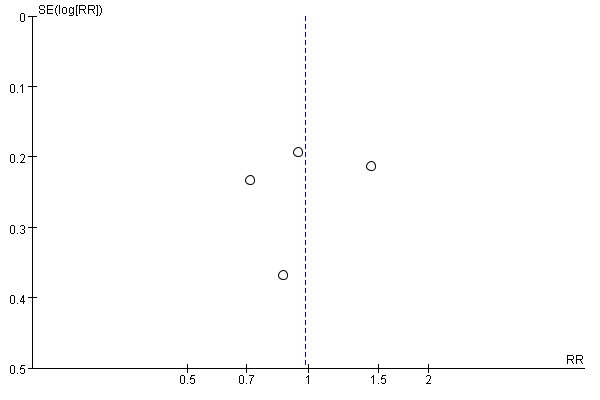


e


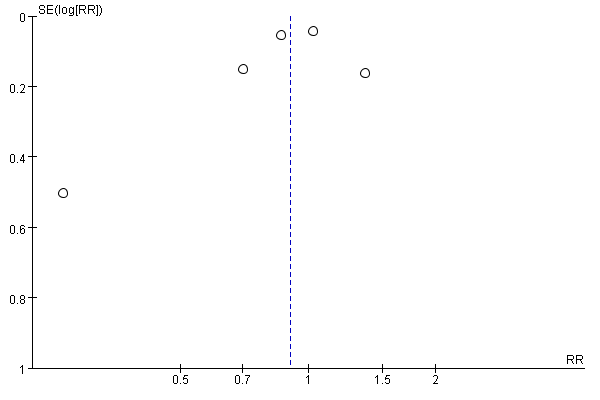


f


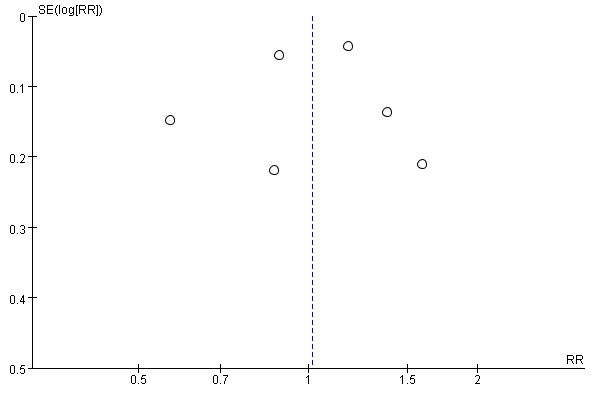


g


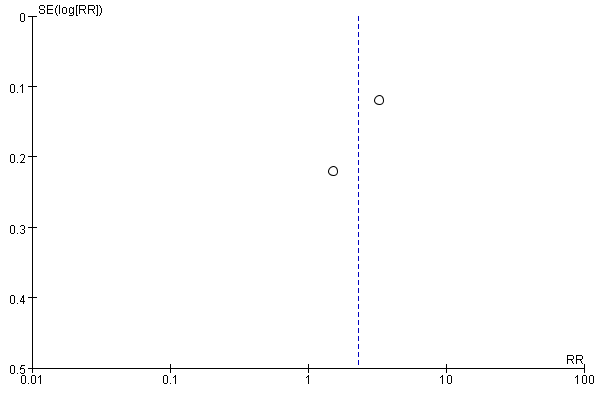


h


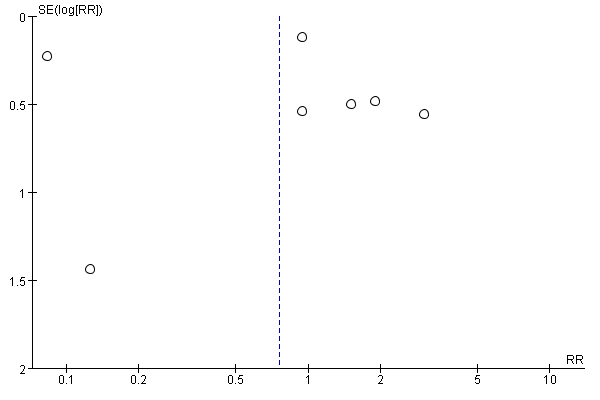


i


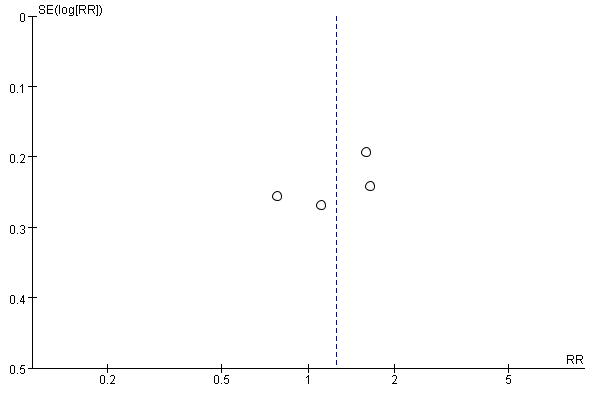


j


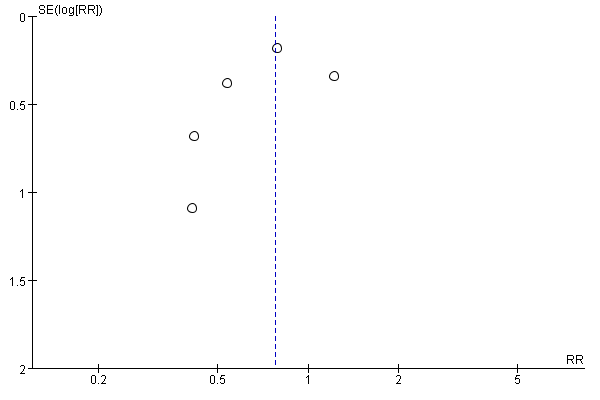


k


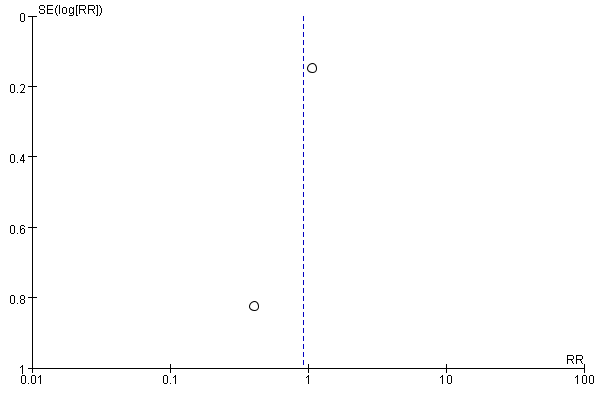


l


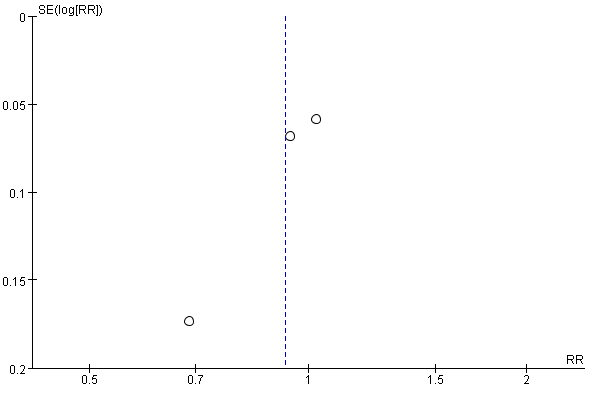


Supplementary Fig. S2: In heterogeneity analysis, miscarriage and premature birth showed low heterogeneity; clinical pregnancy, clinical pregnancy (fresh embryo), clinical pregnancy (frozen embryo), clinical pregnancy (RCT studies), ongoing pregnancy and live birth showed medium heterogeneity; clinical pregnancy (n0n-RCT studies), implantation rate, singleton and multiple pregnancy showed high heterogeneity.

(a) clinical pregnancy, I^2^ = 71%; (b) clinical pregnancy (fresh embryo), I^2^ = 74%; (c) clinical pregnancy (frozen embryo), I^2^ = 74%; (d) clinical pregnancy (RCT studies), I^2^ = 42%; (e) clinical pregnancy (non-RCT studies), I^2^ = 83%; (f) implantation rate, I^2^ = 89%; (g) singleton pregnancy, I^2^ = 89%; (h) multiple pregnancies, I^2^ = 94%; (i) ongoing pregnancy, I^2^ = 54%; (j) miscarriage, I^2^ = 0%; (k) premature birth, I^2^ = 26%; (l)live birth, I^2^ = 63%.
